# Supplementary material for: A Likelihood Approach for Real-Time Calibration of Stochastic Compartmental Epidemic Models
Source: PLoS Comput Biol. 2017 Jan 17;13(1):e1005257. doi: 10.1371/journal.pcbi.1005257 (PMC5240920; doi:10.1371/journal.pcbi.1005257)
Supplement: S1 File — (TAR.GZ) [file pcbi.1005257.s014.tar.gz › HSPH_Online-SI-Revision/PF08/n100-mild/n100-mild_PF_table-8.pdf]

1	R0	{1.24125, 3.38982}	{1.82083, 18.803}	{{1.54545, 15.2869}, {2.09916, 20.}}	{46.6934, 454.69}	inf	218.	1026.24	{704.589, 1469.81}	370.751	0.655283	newly	544.	442.73	{295., 625.}	25.4835	0.135174	Reff	1.2258	1.76449	{1.5227, 2.00028}	43.946	ar	34926.	75983.1	{64923., 84935.}	117.554
2	R0	{1.29554, 4.51878}	{2.01346, 19.1631}	{{1.64682, 15.8595}, {2.13873, 20.}}	{55.4148, 324.078}	inf	496.	1895.18	{1405.84, 2323.9}	282.093	0.574116	newly	1039.	815.32	{660., 997.}	21.9731	0.112893	Reff	1.26727	1.90989	{1.60577, 2.06571}	50.7092	ar	42828.	82505.3	{70252., 86288.}	92.6434
3	R0	{1.2443, 3.64292}	{2.06219, 16.5757}	{{1.61101, 10.3386}, {2.72289, 20.}}	{65.7306, 355.011}	inf	288.	1493.29	{852.093, 2260.64}	418.503	0.68324	newly	581.	783.2	{481., 1058.}	42.0654	0.148059	Reff	1.22536	2.00595	{1.57189, 2.6345}	63.7023	ar	37087.	81585.3	{68613., 95031.}	119.984
4	R0	{1.38465, 3.43331}	{2.07435, 15.8263}	{{1.33605, 10.0969}, {3., 20.}}	{51.36, 360.962}	inf	73.	324.06	{121.519, 649.059}	344.022	0.570866	newly	229.	191.85	{54., 347.}	42.1965	0.242555	Reff	1.38185	2.04315	{1.31261, 2.94766}	49.7469	ar	49511.	75524.5	{45435., 95029.}	56.1051
5	R0	{1.25409, 4.1068}	{1.79373, 15.8862}	{{1.4794, 9.31727}, {2.57163, 20.}}	{43.0305, 286.827}	inf	293.	830.892	{523.785, 1523.85}	183.581	0.420947	newly	618.	438.04	{274., 700.}	35.7605	0.202157	Reff	1.23932	1.74901	{1.42305, 2.50732}	41.1268	ar	38571.	71420.2	{57894., 91489.}	85.1656
6	R0	{1.28821, 4.2016}	{1.89188, 17.6295}	{{1.64893, 14.5325}, {2.14702, 20.}}	{46.8613, 319.589}	inf	415.	1587.43	{1123.91, 1968.91}	282.514	0.571959	newly	847.	745.91	{562., 966.}	17.7721	0.0867516	Reff	1.26157	1.83508	{1.57911, 2.09058}	45.4602	ar	41468.	78920.5	{70040., 86503.}	90.3167
7	R0	{1.24369, 2.56007}	{2.04691, 17.1881}	{{1.50065, 12.7463}, {2.68964, 20.}}	{64.584, 571.393}	inf	56.	426.692	{224.53, 709.24}	661.95	0.835285	newly	202.	219.37	{106., 376.}	42.0248	0.175488	Reff	1.24058	2.02528	{1.48646, 2.68206}	63.2527	ar	38603.	78501.1	{60812., 94246.}	103.355
8	R0	{1.232, 3.53942}	{1.75123, 18.0854}	{{1.52777, 14.117}, {1.93845, 20.}}	{42.1449, 410.972}	inf	337.	1246.46	{937.685, 1522.17}	269.869	0.560212	newly	787.	555.38	{442., 648.}	30.4879	0.161617	Reff	1.2117	1.71917	{1.50057, 1.89896}	41.8814	ar	34555.	74909.6	{63995., 82553.}	116.784
9	R0	{1.36262, 3.85631}	{1.98272, 17.4824}	{{1.53117, 12.6819}, {2.62526, 20.}}	{45.5076, 353.346}	inf	149.	477.177	{261.85, 755.296}	220.45	0.468016	newly	418.	235.84	{120., 359.}	44.2249	0.293026	Reff	1.35645	1.95675	{1.48914, 2.60154}	44.2552	ar	48410.	77270.1	{61384., 92869.}	59.6161
10	R0	{1.28702, 4.67968}	{1.73954, 17.7206}	{{1.56906, 14.1286}, {1.94481, 20.}}	{35.1606, 278.672}	inf	588.	1843.13	{1559.2, 2164.81}	213.458	0.492367	newly	1085.	846.3	{689., 1004.}	22.4129	0.114093	Reff	1.24853	1.68794	{1.52788, 1.89701}	35.1943	ar	41092.	75267.5	{67670., 83571.}	83.1684
11	R0	{1.35752, 3.47129}	{1.83941, 18.4321}	{{1.49398, 13.4049}, {2.11384, 20.}}	{35.4976, 430.986}	inf	93.	575.504	{363.013, 783.434}	518.821	0.771343	newly	286.	257.92	{140., 346.}	22.0839	0.115776	Reff	1.35065	1.80379	{1.48901, 2.07166}	33.5499	ar	44669.	76578.7	{61650., 85872.}	71.4359
12	R0	{1.35929, 5.83647}	{1.8898, 18.0346}	{{1.62957, 15.6698}, {2.16899, 20.}}	{39.0285, 208.998}	inf	673.	2155.55	{1565.36, 2768.39}	220.29	0.494301	newly	1048.	966.97	{752., 1144.}	12.6307	0.0606479	Reff	1.3216	1.79228	{1.59221, 1.99103}	35.6148	ar	46715.	80230.2	{71386., 88501.}	71.744
13	R0	{1.28019, 3.29908}	{1.91695, 18.2099}	{{1.59171, 15.036}, {2.166, 20.}}	{49.7397, 451.97}	inf	93.	509.33	{312.559, 655.508}	447.667	0.713993	newly	305.	239.77	{129., 356.}	31.7672	0.172352	Reff	1.27548	1.89368	{1.58794, 2.15047}	48.4681	ar	40941.	78293.1	{67254., 86569.}	91.2339
14	R0	{1.36661, 3.48318}	{1.95336, 16.0397}	{{1.47358, 9.29535}, {2.63462, 20.}}	{42.9846, 360.491}	inf	101.	419.606	{206.03, 762.615}	315.452	0.567727	newly	296.	237.37	{109., 403.}	39.9696	0.21996	Reff	1.36217	1.92554	{1.45078, 2.61088}	41.404	ar	48672.	75584.3	{58395., 93349.}	55.3462
15	R0	{1.35865, 3.84123}	{1.86253, 18.5726}	{{1.59483, 13.904}, {2.1515, 20.}}	{37.0865, 383.507}	inf	112.	595.401	{364.732, 846.278}	431.608	0.703191	newly	273.	267.84	{142., 381.}	28.8498	0.136204	Reff	1.35217	1.83951	{1.58572, 2.10922}	36.0415	ar	48598.	77327.8	{66743., 86170.}	59.1172
16	R0	{1.22879, 3.98329}	{1.76385, 18.4323}	{{1.57316, 14.6391}, {1.9145, 20.}}	{43.5433, 362.742}	inf	457.	1572.98	{1219.25, 1871.3}	244.196	0.530607	newly	913.	687.76	{562., 888.}	25.5465	0.132347	Reff	1.20087	1.71475	{1.5379, 1.87334}	42.7923	ar	34405.	76104.3	{67486., 82381.}	121.201
17	R0	{1.34925, 3.64524}	{1.94083, 16.5393}	{{1.47794, 12.3371}, {2.63619, 20.}}	{43.8452, 353.722}	inf	135.	499.496	{297.072, 801.563}	269.997	0.531353	newly	291.	260.2	{140., 381.}	30.6667	0.154168	Reff	1.34265	1.91529	{1.45279, 2.62237}	42.6498	ar	46871.	75577.7	{59525., 92898.}	61.2461
18	R0	{1.33054, 2.25275}	{1.94799, 13.5209}	{{1.18976, 6.16453}, {2.75598, 19.5242}}	{50.6571, 500.194}	inf	11.	164.002	{16.5687, 388.95}	1403.48	1.03894	newly	52.	131.74	{6., 285.}	190.	0.542279	Reff	1.33009	1.90157	{1.15086, 2.74816}	48.2983	ar	44336.	65116.9	{20694., 91955.}	71.8358
19	R0	{1.28394, 3.69859}	{1.78741, 19.1064}	{{1.55072, 14.7721}, {1.91769, 20.}}	{39.2131, 416.585}	inf	236.	1243.8	{1005.89, 1492.5}	427.033	0.715721	newly	569.	529.29	{403., 689.}	16.2091	0.0748117	Reff	1.26314	1.75149	{1.52705, 1.88982}	38.6614	ar	39558.	76996.9	{66071., 82651.}	94.643
20	R0	{1.2883, 2.26493}	{1.92155, 13.8392}	{{1.19018, 6.16453}, {2.75598, 19.5242}}	{52.1252, 511.022}	inf	9.	167.209	{16.5687, 362.69}	1770.4	1.128	newly	36.	116.88	{6., 271.}	253.389	0.615216	Reff	1.28811	1.87201	{1.15086, 2.7524}	49.6441	ar	40768.	63721.2	{27943., 91955.}	78.7971
21	R0	{1.34098, 2.78621}	{1.95108, 13.9349}	{{1.19018, 6.18069}, {2.85956, 19.5242}}	{49.8005, 400.139}	inf	10.	164.487	{16.5687, 362.69}	1556.5	1.07229	newly	66.	120.52	{6., 261.}	124.121	0.482228	Reff	1.34068	1.90326	{1.15086, 2.7524}	47.5051	ar	44605.	65992.	{28247., 92658.}	70.0297
22	R0	{1.33783, 5.95911}	{1.94696, 18.2595}	{{1.62445, 14.4771}, {2.16105, 20.}}	{45.531, 206.413}	inf	837.	2511.94	{1829.4, 3150.35}	200.112	0.468085	newly	1123.	1120.53	{951., 1294.}	10.4265	0.0453908	Reff	1.29617	1.82179	{1.56884, 2.00193}	40.5516	ar	45418.	81645.	{70897., 87922.}	79.7636
23	R0	{1.33359, 3.3069}	{1.78981, 16.8296}	{{1.45333, 12.7116}, {2.05958, 20.}}	{34.2099, 408.923}	inf	97.	488.76	{298.468, 746.849}	403.876	0.671002	newly	260.	246.15	{136., 386.}	30.5962	0.145469	Reff	1.32804	1.75102	{1.41678, 2.00606}	31.8493	ar	46245.	71817.8	{56171., 82479.}	55.2985
24	R0	{1.30588, 2.56123}	{2.02924, 15.7353}	{{1.3704, 8.45965}, {2.86816, 20.}}	{55.5455, 514.366}	inf	22.	256.933	{70.8202, 504.992}	1067.88	0.959268	newly	124.	169.9	{39., 344.}	80.129	0.286037	Reff	1.30531	2.00322	{1.36923, 2.8473}	53.6744	ar	42383.	74969.7	{50407., 93929.}	77.2706
25	R0	{1.29813, 4.51849}	{1.84997, 19.9147}	{{1.72439, 20.}, {1.91464, 20.}}	{42.5106, 340.737}	inf	361.	1608.21	{1343.95, 1839.84}	345.487	0.645577	newly	633.	651.94	{529., 751.}	11.8325	0.0503206	Reff	1.27199	1.80403	{1.68318, 1.85655}	41.8282	ar	43371.	79725.1	{75280., 82192.}	83.8213
26	R0	{1.33231, 3.99953}	{1.81364, 18.282}	{{1.57544, 14.5956}, {2.1144, 20.}}	{36.1272, 357.104}	inf	222.	1115.28	{801.716, 1624.62}	402.377	0.686975	newly	479.	499.09	{326., 692.}	23.2463	0.0980094	Reff	1.3147	1.78095	{1.56235, 2.02398}	35.4646	ar	44733.	77727.6	{67704., 87343.}	73.759
27	R0	{1.30555, 4.61445}	{1.81515, 19.9905}	{{1.76691, 20.}, {1.86748, 20.}}	{39.0332, 333.215}	inf	301.	1224.2	{1029.82, 1428.64}	306.713	0.606357	newly	599.	491.93	{407., 579.}	18.7362	0.092807	Reff	1.2885	1.78128	{1.73706, 1.83346}	38.2449	ar	42624.	78489.3	{76708., 80412.}	84.1433
28	R0	{1.26819, 5.34969}	{1.86944, 18.3933}	{{1.58707, 15.3058}, {2.10891, 20.}}	{47.4104, 243.819}	inf	559.	1607.49	{1067.6, 2058.91}	187.564	0.445469	newly	884.	707.07	{512., 853.}	20.793	0.108723	Reff	1.2407	1.7846	{1.55665, 2.01957}	43.839	ar	38946.	77588.9	{67700., 85486.}	99.2217
29	R0	{1.19925, 3.00394}	{1.88103, 17.4647}	{{1.58962, 14.7978}, {2.13709, 20.}}	{56.8504, 481.392}	inf	185.	1028.38	{741.006, 1301.84}	455.879	0.734529	newly	516.	493.45	{375., 648.}	18.5019	0.0852915	Reff	1.18521	1.83069	{1.55535, 2.07396}	54.4618	ar	31727.	77972.1	{67180., 86177.}	145.759
30	R0	{1.35749, 3.75483}	{1.74307, 17.6997}	{{1.46596, 13.3487}, {2.02195, 20.}}	{28.4055, 371.386}	inf	91.	391.554	{219.519, 526.454}	330.279	0.609743	newly	259.	184.52	{85., 262.}	33.444	0.199587	Reff	1.35287	1.71605	{1.41457, 1.98975}	26.8496	ar	48244.	71141.8	{57775., 81912.}	47.4626
31	R0	{1.27131, 4.2161}	{1.76159, 18.5921}	{{1.60098, 14.4681}, {1.91815, 20.}}	{38.5653, 340.979}	inf	214.	880.797	{676.309, 1014.03}	311.587	0.607329	newly	451.	385.11	{287., 485.}	20.4678	0.10164	Reff	1.25808	1.7442	{1.58475, 1.90144}	38.6401	ar	38299.	75909.9	{68288., 82501.}	98.2033
32	R0	{1.31827, 6.18119}	{1.90109, 18.0735}	{{1.66015, 14.2807}, {2.14437, 20.}}	{44.2107, 192.394}	inf	869.	2326.7	{1691.63, 2843.71}	167.745	0.418508	newly	1107.	1049.76	{844., 1222.}	9.88799	0.0463078	Reff	1.27462	1.80063	{1.59712, 2.00167}	41.268	ar	45262.	80908.6	{72075., 88353.}	78.7562
33	R0	{1.20398, 1.35167}	{1.94663, 13.9055}	{{1.19018, 6.16453}, {2.85956, 19.5242}}	{62.4177, 928.765}	inf	3.	173.231	{16.5687, 388.95}	5681.91	1.54035	newly	20.	132.84	{6., 270.}	587.1	0.773682	Reff	1.20375	1.89779	{1.15086, 2.7524}	59.3185	ar	33329.	65620.4	{28247., 92658.}	111.507
34	R0	{1.32776, 3.146}	{2.05023, 18.2139}	{{1.50828, 14.8748}, {2.65323, 20.}}	{54.413, 478.953}	inf	86.	453.326	{223.291, 771.76}	427.123	0.675013	newly	292.	218.48	{90., 356.}	37.4726	0.221359	Reff	1.32356	2.03303	{1.49634, 2.63786}	53.6029	ar	44872.	78838.1	{60983., 93231.}	75.6956
35	R0	{1.279, 1.11336}	{1.96497, 13.6494}	{{1.20956, 6.18069}, {2.85956, 19.5412}}	{55.9205, 1125.96}	inf	8.	183.037	{16.5687, 407.329}	2198.6	1.18596	newly	51.	129.69	{6., 295.}	187.314	0.545417	Reff	1.27844	1.92289	{1.15086, 2.7524}	53.5982	ar	41033.	66018.5	{27943., 92658.}	81.4042
36	R0	{1.20029, 1.94717}	{1.92188, 13.8656}	{{1.18976, 6.16453}, {2.75598, 19.5242}}	{60.9383, 612.089}	inf	9.	165.099	{16.5687, 362.69}	1746.96	1.12643	newly	29.	116.02	{6., 261.}	326.621	0.672105	Reff	1.20009	1.87217	{1.15086, 2.7524}	57.8947	ar	33234.	63577.7	{17527., 91955.}	109.086
37	R0	{1.33216, 3.69685}	{1.85808, 18.2408}	{{1.54809, 15.0813}, {2.12541, 20.}}	{39.4788, 393.413}	inf	191.	913.28	{566.568, 1180.52}	378.157	0.664171	newly	501.	412.34	{248., 546.}	22.5509	0.121263	Reff	1.3191	1.81466	{1.53603, 2.06224}	37.5676	ar	45235.	77424.1	{66043., 85644.}	71.1597
38	R0	{1.25434, 2.04314}	{1.92437, 13.5196}	{{1.18976, 6.16453}, {2.75598, 19.5242}}	{55.645, 561.709}	inf	23.	160.664	{16.5687, 362.69}	615.612	0.778805	newly	145.	122.18	{6., 271.}	68.7586	0.546629	Reff	1.25374	1.87896	{1.15086, 2.74816}	53.3411	ar	38662.	63901.1	{19179., 91955.}	87.7502
39	R0	{1.27466, 3.0741}	{1.88949, 14.9395}	{{1.3068, 7.84386}, {2.75521, 20.}}	{48.2924, 385.979}	inf	125.	441.894	{183.847, 692.002}	253.515	0.500773	newly	445.	262.79	{121., 455.}	43.6427	0.286349	Reff	1.2688	1.84437	{1.28959, 2.73128}	45.501	ar	40181.	69983.2	{44685., 93101.}	74.3847
40	R0	{1.33045, 2.68154}	{2.05602, 15.4149}	{{1.37883, 10.9996}, {2.96527, 20.}}	{55.0129, 474.854}	inf	25.	274.519	{87.9494, 540.836}	998.074	0.946008	newly	85.	181.37	{42., 376.}	135.588	0.323608	Reff	1.32907	2.01849	{1.35121, 2.92047}	52.4601	ar	45951.	75567.8	{50704., 94826.}	65.7459
41	R0	{1.37003, 2.83984}	{1.93333, 13.9106}	{{1.19018, 6.18069}, {2.75598, 19.5242}}	{46.5429, 389.838}	inf	9.	168.146	{16.5687, 388.95}	1779.22	1.1159	newly	52.	121.47	{6., 261.}	168.635	0.521616	Reff	1.36976	1.88468	{1.15086, 2.74816}	44.4189	ar	48926.	65336.	{28247., 91955.}	58.9421
42	R0	{1.3003, 2.94885}	{1.9286, 16.4074}	{{1.49406, 9.38661}, {2.62802, 20.}}	{48.3193, 456.401}	inf	51.	317.825	{185.48, 531.735}	523.187	0.750928	newly	214.	173.75	{80., 284.}	36.9112	0.20436	Reff	1.29797	1.91015	{1.46278, 2.61411}	47.1642	ar	44237.	75203.	{60079., 92954.}	70.0003
43	R0	{1.33756, 2.92653}	{1.95315, 13.7131}	{{1.19018, 6.16288}, {2.75598, 19.5242}}	{50.1461, 368.58}	inf	19.	168.767	{16.5687, 388.95}	801.876	0.846758	newly	61.	136.34	{9., 285.}	162.164	0.494904	Reff	1.33705	1.9047	{1.15086, 2.74816}	47.6416	ar	47342.	66266.7	{28247., 91955.}	63.4029
44	R0	{1.35801, 2.92276}	{1.96757, 14.8881}	{{1.31613, 7.76251}, {2.96546, 20.}}	{46.1444, 409.385}	inf	11.	222.069	{81.0613, 393.215}	1918.81	1.22512	newly	66.	168.71	{49., 333.}	168.318	0.332908	Reff	1.35749	1.94072	{1.30063, 2.94302}	44.5099	ar	47940.	71767.	{44386., 94839.}	52.9084
45	R0	{1.31342, 4.02649}	{2.01126, 16.7531}	{{1.49442, 9.28872}, {2.64577, 20.}}	{53.1318, 316.073}	inf	222.	866.826	{447.168, 1311.74}	290.462	0.548725	newly	477.	446.8	{238., 634.}	28.9392	0.143478	Reff	1.30142	1.97566	{1.47126, 2.5924}	51.8088	ar	42995.	77464.8	{61000., 92949.}	80.1717
46	R0	{1.37919, 3.75889}	{1.98893, 18.1067}	{{1.53922, 13.6869}, {2.6249, 20.}}	{44.2239, 381.702}	inf	78.	421.458	{189.626, 712.799}	440.331	0.691546	newly	193.	202.12	{77., 337.}	37.5751	0.169237	Reff	1.37534	1.96208	{1.50903, 2.59915}	42.6754	ar	49157.	77792.5	{62619., 92839.}	58.2531
47	R0	{1.37458, 3.56117}	{1.91805, 17.1227}	{{1.48878, 12.7981}, {2.59986, 20.}}	{39.537, 380.816}	inf	90.	383.939	{216.676, 669.09}	326.599	0.59203	newly	236.	191.73	{89., 322.}	35.6822	0.194361	Reff	1.37118	1.89453	{1.48282, 2.57143}	38.1672	ar	48478.	75370.9	{59937., 92557.}	55.4744
48	R0	{1.33393, 3.78296}	{1.80701, 19.9955}	{{1.72206, 20.}, {1.87129, 20.}}	{35.4652, 428.567}	inf	172.	946.705	{789.512, 1091.11}	450.41	0.737196	newly	441.	378.86	{311., 442.}	15.7778	0.0771501	Reff	1.3221	1.78495	{1.70574, 1.84687}	35.009	ar	44885.	78204.7	{74941., 80629.}	74.2334
49	R0	{1.24918, 2.39046}	{1.88088, 15.8661}	{{1.30984, 8.13172}, {2.75948, 20.}}	{50.6211, 563.726}	inf	41.	393.623	{171.878, 623.504}	860.057	0.929319	newly	241.	219.28	{78., 349.}	39.5602	0.200358	Reff	1.2448	1.84473	{1.29096, 2.73147}	48.294	ar	35969.	69270.	{44724., 93145.}	92.5826
50	R0	{1.28281, 2.3032}	{1.96503, 15.7537}	{{1.37124, 8.42245}, {2.95383, 20.}}	{53.3047, 583.991}	inf	28.	322.705	{104.552, 591.317}	1052.52	0.978714	newly	129.	183.46	{68., 311.}	67.3488	0.233779	Reff	1.28046	1.93654	{1.3366, 2.92473}	51.4075	ar	40223.	72476.5	{48645., 94630.}	80.4881
